# Generalized maximum entropy: when and why you need it

**DOI:** 10.1098/rsos.260003

**Published:** 2026-11-04

**Authors:** Giuseppe M. Ferro, Andrea Somazzi, Edwin Pos

**Affiliations:** ^1^ Center for Statistics and Machine Learning, Princeton University, Princeton, NJ, USA; ^2^ Department of Ecology and Evolutionary Biology, Princeton University, Princeton, NJ, USA; ^3^ IMT School for Advanced Studies Lucca, Lucca, Italy; ^4^ Quantitative Biodiversity Dynamics, Ecology and Biodiversity, Utrecht University, Utrecht, The Netherlands; ^5^ Botanic Gardens, Utrecht University, Utrecht, The Netherlands

**Keywords:** maximum entropy principle, statistical inference, complex systems

## Abstract

The classical maximum entropy principle based on Shannon entropy is widely used to construct least-biased probability distributions from partial information. However, the Shore–Johnson axioms that single out the Shannon functional hinge on *strong system independence*—an assumption often violated in real-world, strongly correlated systems. When that assumption fails, enforcing the Shannon form imposes an independence the data do not support, and can systematically distort the inferred distribution. We provide a self-contained guide to *when and why* practitioners should abandon the Shannon form in favour of the one-parameter Uffink–Jizba–Korbel (UJK) family of generalized entropies. After reviewing the Shore–Johnson axioms from an applied perspective, we recall the most commonly used entropy functionals and locate them within the UJK family. The need for generalized entropies is illustrated with two applications, one in economics and the other in ecology. A simple mathematical model worked out in detail further shows the power of generalized maximum-entropy approaches in cases where strong system independence does not hold. We conclude with practical guidelines for choosing an entropy measure and reporting results so that analyses remain transparent and reproducible.

## 1. Introduction

In several disciplines of science, ranging from economics and ecology to physics and the social sciences, entropy measures are considered crucial for describing and modelling complex systems [1]. One particular application concerns deriving the most likely probability distribution related to a pattern of interest based on prior information [2,3] (box 1). These patterns can be as varied as the position and momentum of gas molecules, the concentration of wealth among countries or age categories or even the distribution of individuals over species. In each case, the key step is to connect macrostate variables—coarse-grained descriptors of the system—to the underlying distribution of microstates. This connection allows us either to infer causal mechanisms or to use the inferred distribution as input for modelling the system as a whole [4]. The framework of entropy maximization allows for a more objective understanding based on a top-down approach using only available data instead of incorporating prior beliefs based on assumptions (as is the case for many parametric methods). The maximum (information) entropy principle (MEP), stemming from the fields of statistical mechanics and information theory, allows us to derive the least-biased probability distribution that represents our current state of knowledge. It does so by maximizing the information entropy of a distribution of interest while respecting certain known constraints, expressed often in the form of expected values using the principle of Lagrange multipliers, which reflect prior information [5].

Traditionally, Shannon entropy is used for the purpose of quantifying the uncertainty (i.e. entropy) of a derived probability distribution. Given a discrete random variable X that can assume N states and denoting by $p_{i}$ the probability for X of being in state i, the formulation of entropy here is in the form of information, the summation of the probability $p_{i}$ of observing i out of N possibilities multiplied by the logarithm of that probability, with base 2 being the traditional application: $H=-\sum_{i=1}^{N}p_{i}\log(p_{i})$ [6]. As said above, the maximization of (Shannon) entropy is useful because it allows a least-biased prediction. It has been also shown that Shannon entropy is the only functional form that satisfies ‘reasonable’ properties, or axioms, from an inferential point of view [7]. However, because many real-world systems do not adhere to such axiomatic structure (further outlined below), there has been considerable effort in relaxing those axioms, leading to a body of literature together called generalized measures of entropy [8]. While the relative merits of different entropy forms have been the subject of much debate in statistical inference, practical criteria for choosing among them are still lacking. This gap is particularly important now, as interest in applying MEP to complex systems continues to rise. Our contribution is to provide both a conceptual framework—by revisiting the Shore–Johnson axioms—and a practical workflow that indicates when generalized entropies are needed and how to implement them. This workflow, developed in §6, consolidates guidance that is otherwise scattered across highly technical literature.

To provide the broader scope in the field of generalized entropies, in §2, we provide a conceptual overview of the axioms of maximum entropy as derived by Shore & Johnson [7]. In §3, we summarize various often-used quantitative measures of entropy. To show why a generalized treatment is necessary, §4 develops two concrete case studies—one drawn from economics, the other from ecology—that expose the practical hurdles of the standard approach. Readers who want a worked derivation can turn to §5, where we apply the generalized MEP to a minimal yet sufficiently rich model; this section may be skipped on a first reading. Finally, §6 discusses the theoretical and practical implications of the generalized framework and offers guidelines for selecting an appropriate entropy measure. Section 7 concludes. The appendices complement the main text with additional mathematical derivations.

**Box 1. Classical maximum-entropy recipe**

**Problem.** Let Ω be a finite or countable set of microstates x. For m observables $f_{i}:\Omega \;\to \;R$, we know their sample means

$$\langle f_{i}\rangle \;=\;\sum_{x\in \Omega }p_{x}f_{i}(x)\;=\;F_{i},\;i=1,\ldots ,m.$$

Determine the *least-biased*^*a*^ probability distribution $P^{\,}=\{p_{x}\}$. **Solution.** Maximize Shannon entropy

$$H(P)=-\sum_{x\in \Omega }p_{x}\ln p_{x}$$

under the (m+1) constraints $\sum_{x}p_{x}=1$ and $\sum_{x}p_{x}f_{i}(x)=F_{i}$. Introduce Lagrange multipliers $\lambda_{0},\lambda_{1},\ldots ,\lambda_{m}$ and set the derivatives of the Lagrangian to zero:

$$L=-\sum_{x}p_{x}\ln p_{x}+\lambda_{0}(\sum_{x}p_{x}-1)+\sum_{i=1}^{m}\lambda_{i}(\sum_{x}p_{x}f_{i}(x)-F_{i}).$$

Stationarity yields the *exponential-family (Gibbs) form*

$$p_{x}^{\,}=\frac{\exp\;\left(-\sum_{i=1}^{m}\lambda_{i}f_{i}(x)\right)}{Z\left(\lambda \right)},\;Z(\lambda )=\sum_{x\in \Omega }\exp\;\left(-\sum_{i=1}^{m}\lambda_{i}f_{i}(x)\right).$$

The multipliers are determined by the dual system

$$F_{i}\;=\;-\;\frac{\partial \ln Z}{\partial \lambda_{i}},\;i=1,\ldots ,m,$$

which can be solved numerically (e.g. Newton or gradient descent) or analytically for simple cases. **Takeaway.** Once the constraints $F_{i}$ and observables $f_{i}$ are specified, *all* that remains is solving for λ; the resulting $P^{\,}$ is automatically the maximum-entropy distribution.

^
*a*
^
In the Shannon sense of preserving only the stated constraints (Shore–Johnson consistency).

## 2. The axioms from a practical point of view

The when and why of generalized maximum entropy can be summarized in terms of assumptions that may or may not be violated, similar to how certain assumptions are violated for parametric statistical methods that then give the necessity of non-parametric approaches [9]. In the early developments, Jaynes already suggested that there exist such axiomatic conditions (although they were not named as such) that were later summarized and derived more thoroughly by Shore & Johnson [7] as well as others (e.g. [10–12]). The axioms to single out Shannon entropy for the MEP can be described as the following:

— Axiom 1: uniqueness: the result should be unique.
— Axiom 2: permutation invariance: state permutation should not matter.
— Axiom 3: subset independence: it should not matter whether one treats disjoint subsets of system states in terms of separate conditional distributions or in terms of the full distribution.
— Axiom 4a: system independence (SI): it should not matter whether one accounts for independent constraints related to independent systems separately in terms of marginal distributions or in terms of full-system constraints and joint distributions.
— Axiom 5: maximality: in absence of any prior information, a uniform distribution should be the solution.

In the original derivation by Shore & Johnson [7], however, the formal step used to derive the Shannon functional corresponds to a *stronger* condition that we will call **strong system independence (SSI)**:

— Axiom 4b: SSI: whenever two subsystems of a system are disjoint, we can treat the subsystems in terms of independent distributions.

Whereas axioms 1, 2, 3 and 5 are generally satisfied by almost any form of entropy measure used, axiom 4b potentially leads to distinct limitations in the use of MEP as statistical inference [11]. Shannon entropy maximization under independent constraints leads to a joint distribution that factorizes. However, when using generalized entropy measures like Rényi entropy, even with independent constraints, the resulting joint distribution may not factorize, revealing the potential correlations between subsystems. This is explained in more detail below, but the divergence between these two examples highlights the limitations of Shannon entropy in capturing potential dependencies that may arise from the intrinsic properties of the entropy measure itself.

Because these axioms may be unfamiliar, we (i) provide practical descriptions of each axiom below and (ii) include in §2.4 a concise box that highlights the key structural implication of *SSI*: it enforces exponential growth of the phase space with system size. By contrast, many complex systems exhibit sub- or super-exponential growth, in which case Shannon entropy is not an adequate inference functional. Generalized entropies become relevant in such cases, as they remain agnostic with respect to the specific growth law of the phase space.

*Summary.* Shannon’s standard MEP corresponds to axioms 1, 2, 3, 5 *and* the *strong* version 4b (SSI). Some systems may violate axiom 4b. If one assumes only the *weak* 4a (SI) rather than 4b, a broader one-parameter family of generalized entropies is admissible; Shannon is recovered as the special case where SSI (and exponential phase-space growth) happens to hold.

### 2.1. Axiom 1: uniqueness

The first axiom states there should only be one single solution that (i) ensures the maximization of the information entropy of the derived distribution and (ii) adheres to the given constraints from prior information. The intuitive explanation is straightforward: if the procedure of entropy maximization resulted in multiple solutions that could be considered equally likely, MEP would fail as a robust inference procedure as we would have no idea which solution is the best and provides the least-biased result. When applying MEP to derive a probability distribution under known constraints from prior knowledge, this axiom ensures we will arrive at the same result, no matter what.

### 2.2. Axiom 2: permutation invariance

The MEP procedure is permutation-invariant: relabelling or reordering the states must not affect the resulting distribution. The inference should depend only on the structure of the problem, not on arbitrary naming conventions. Of course, if the states are relabelled, the corresponding constraints must be relabelled in the same way; otherwise the problem itself has changed.

### 2.3. Axiom 3: subset independence

This axiom deals with the fact that whether we were to look at either the complete ensemble of states in terms of configuration space or at subsets of states comprising that complete ensemble should not matter. This means that either specifying the full system distribution or separate conditional distributions for states should not make a difference; the system itself is in some sense decomposable in its constituent elements.

### 2.4. Axiom 4: system independence versus strong system independence

This axiom concerns multiple systems—distinct entities that together form a larger composite system but that are independent in the sense of being separated in space, time or causal interaction. Crucially, this is not about partitions of a single state space (axiom 3): it concerns the relationship between two genuinely separate systems, each with its own state space and constraints.

SI (axiom 4a) requires that new information about one system should not alter the inferred distribution of the other. In other words, updating the marginal distribution of system A must leave the marginal of system B unchanged, provided the systems are assumed independent. The precise requirement is only that inferring the two systems separately and jointly never conflict; its formal statement and the exact sense in which it is weaker than SSI are given in appendix A.

SSI (axiom 4b) adds the stronger demand that the joint posterior distribution remains a product measure under independent constraints. With Shannon entropy, this implies strict additivity H[AB]=H[A]+H[B] and, as shown in box 2, enforces exponential phase-space growth. Generalized entropies (e.g. Rényi/Tsallis) satisfy SI but *need not* satisfy SSI; their max entropy joints can exhibit correlations even when constraints are independent. For the technical reader, we give the formal statement of both axioms in appendix A.

**Box 2. SSI enforces exponential growth of the phase space**

**Setting (system size N versus sample length k).** A single configuration of a size-N system is $\sigma \in \Omega^{\left(N\right)}$ with probability $p_{N}(\sigma )$. When we write a sequence $\Sigma^{\left(k\right)}=(\sigma^{\left(1\right)},\ldots ,\sigma^{\left(k\right)})$, we mean k *independent* draws from $p_{N}$ (i.i.d. configurations of length N). **Independent constraints and SSI.** Consider two disjoint subsystems A and B with state spaces $\Omega_{A},\Omega_{B}$ and maximum-entropy posteriors $p_{A}^{⋆},p_{B}^{⋆}$ obtained from constraints that *do not couple* A and B. *SSI* requires the joint posterior to factorize:

$$p_{AB}^{⋆}(x,y)=p_{A}^{⋆}(x)\;p_{B}^{⋆}(y).$$

**Shannon additivity for products.** For any product measure,

$$H[p_{AB}^{⋆}]=H[p_{A}^{⋆}]+H[p_{B}^{⋆}],\;H[p]:=-\sum p\log p.$$

**Asymptotic equipartition property (AEP).** Let $\Sigma^{\left(k\right)}$ be k i.i.d. draws from $p_{N}$. Then, by the law of large numbers applied to $-\log p_{N}(\sigma )$,

$$-\frac{1}{k}\log p_{N}^{⊗k}\;(\Sigma^{\left(k\right)})\;\overset{P\;}{\underset{k\to \infty \;}{\to }}\;H[p_{N}].$$

Hence, for large k, almost all sequences have probability $\approx e^{-kH[p_{N}]}$, so the *typical set* of sequences has size

$$|A^{\left(k\right)}|\;\approx \;e^{kH[p_{N}]}.$$

This shows that $H[p_{N}]$ measures the log of the effective number of configurations *per draw*. Accordingly, for a single configuration of size N, we define^*a*^

$$W_{\mathrm{typ}}(N):=e^{H[p_{N}]}.$$

**From SSI to multiplicativity of typical counts.** Applying AEP to $p_{A}^{⋆}$, $p_{B}^{⋆}$, and their product $p_{AB}^{⋆}$, and using additivity:

$$\begin{array}{rl} W_{\mathrm{typ}}(N\;+\;M) & =e^{H[p_{AB}^{⋆}]}=e^{H[p_{A}^{⋆}]+H[p_{B}^{⋆}]}\\ & =W_{\mathrm{typ}}(N)\;W_{\mathrm{typ}}(M). \end{array}$$

Therefore $W_{\mathrm{typ}}$ satisfies the Cauchy multiplicative equation, whose positive solutions are exponential:

$$W_{\mathrm{typ}}(N)=\mu^{\;N},\;\mu >1.$$

**Takeaway.**

SSI (factorization)⇒H additivity⇒typical-set multiplicativity (AEP)⇒Wtyp(N)=μN.

**Remark.** The AEP always uses the Shannon entropy of the distribution $p_{N}$, regardless of which entropy functional (Shannon, Rényi, Tsallis, etc.) was used to derive $p_{N}^{⋆}$. These are distinct roles of ‘entropy’.

^
*a*
^
If $p_{N}$ is uniform on W(N) states, then $H[p_{N}]=\log W(N)$ and $W_{\mathrm{typ}}(N)=e^{H[p_{N}]}=W(N)$. The AEP shows this relationship extends to general (non-uniform) $p_{N}$: S still counts the logarithm of the *effective* number of states.

### 2.5. Axiom 5: maximality

The principle of maximum entropy is strongly related to the principle of indifference, dating back centuries at the beginning of probability thinking. This principle states that in the absence of alternative evidence, all possible outcomes should be considered equally likely. The maximization of entropy thus means that in the absence of any prior information a uniform distribution of probability over all possible states should be the solution. In slightly more mathematical terms, if we have system with $x_{1}$ to $x_{n}$ possible states but we have absolutely no prior information on how likely each would be, then the most uninformed prediction would simply be that $P(x_{i})=1/n$ for all x. In the spirit of the principle of indifference, the maximization of entropy ensures that we predict a distribution that only incorporates information we do have instead of introducing assumptions for which we have no evidence.

## 3. A family of entropies

Considering the above axioms, the only functional form of the entropy satisfying axioms 1, 2, 3, 5 and 4b is Shannon entropy [7]. However, by relaxing axiom 4b from SSI (formally used by Shore & Johnson [7] in their proof) to SI (axiom 4a), a one-parameter generalized family of entropies emerges as suitable for maximization in an inference procedure [11].

As mentioned in the previous section, the axiom of *SI* states it should not matter whether one accounts for independent constraints related to independent systems separately in terms of marginal distributions or in terms of full-system constraints and joint distribution. Such relaxed version of the axiom states that the inference procedure has to remain coherent at all the scales MEP can be applied, but does not assume that independent information automatically translates into factorizable probabilities when both prior distributions and pieces of information are independent. Clearly, having no information about interaction encoded in constraints (i.e. having independent constraints) is not the same as having no correlations among systems. This axiom leads then to a generalized entropic family [11,13]. For a probability distribution $P=\{p_{i}\}$ and a real *entropic index* q, it is obtained by applying a monotonically increasing function f to the functional $U_{q}(P)=(\sum_{i}p_{i}^{q}{)}^{1/(1-q)}$:

(3.1) $$H_{q}^{\left(f\right)}=f(U_{q}(P))=f\;\left[(\sum_{i}p_{i}^{q}{)}^{1/(1-q)}\right].$$

When *SSI* is enforced, the only possible q is q=1, which results in the original Shannon entropy. Other notable entropies that are members of this family are Rényi entropy (corresponding to f(x)=ln⁡x) and Tsallis entropy (corresponding to $f(x)=\ln_{q}x=(x^{1-q}-1)/(1-q)$) (see below). Note that as f is monotonically increasing, all these entropies share the same maximizing probability distribution. There exist many different functional forms of entropy, and in the following, we describe four often encountered forms for the practical user: Shannon entropy, relative Shannon entropy, Rényi entropy and Tsallis entropy.

### 3.1. Shannon entropy

Shannon entropy, introduced in [6], is defined as

(3.2) $$H(X)=-\sum_{i\in \chi }p_{i}\ln p_{i}.$$

It quantifies the expected amount of information—or equivalently, the average level of uncertainty—contained in a probability distribution. It is additive for independent systems, i.e. H(X,Y)=H(X)+H(Y) if X and Y are independent random variables. It is also worth mentioning that the Shannon entropy of a uniform probability distribution U is the Boltzmann entropy H(U)=ln⁡Ω, where Ω is the volume of the state space (i.e. how many states the random variable can have).

### 3.2. Relative Shannon entropy

For two discrete probability distributions p and q over the same sample space χ, the relative Shannon entropy (Kullback–Leibler divergence) of p relative to q is defined as [14]

(3.3) $$D_{\text{KL}}(p||q)=\sum_{i\in \chi }p_{i}\ln\frac{p_{i}}{q_{i}}.$$

Relative Shannon entropy measures how much a probability distribution diverges from a second one. In inference and statistical modelling, it is the proper function to maximize to infer the distribution p under certain constraints, when the prior distribution is q. In this paper, we will focus in the standard maximum-entropy approach, thus assuming the prior q uniform.

### 3.3. Rényi entropy

Rényi entropy is defined as [15]

(3.4) $$R_{q}(X)\equiv H_{q}^{\ln}=\frac{1}{1-q}\ln(\sum_{i\in \chi }p_{i}^{q}).$$

Note that different values of q place different emphases on the tail behaviour of the distribution. Because of this flexibility, Rényi entropy appears in diverse areas such as ecology, coding and various inference-based disciplines.

Ecologists often evaluate and convert Rényi entropy to Hill numbers, sometimes called the effective number of species, via the exponential transform ${}^{q}D=\exp(R_{q}(X))$ which directly yields an intuitive idealized ‘number of equally common species’ interpretation. Varying q produces a Rényi diversity profile that shows how a community’s diversity changes based on the relative emphasis on rare versus abundant species [16,17]. Note that from the point of view of entropy maximization, it has been proven that Rényi entropy is the only member of the family (3.1) to retrieve consistency between the maximum entropy and the maximum likelihood principles [18]. Notable Rényi entropy properties are additivity for independent random variable (same as Shannon entropy), non-decreasing function of q, and that it recovers Shannon entropy:

(3.5) $$\lim_{q\to 1}R_{q}(X)=H(X).$$

Finally, for a uniform distribution U, Rényi entropy is independent of q and equal to Boltzmann entropy, i.e. $R_{q}(U)=\ln\Omega$.

### 3.4. Tsallis entropy

Tsallis entropy is defined as [19,20]

(3.6) $$T_{q}(X)\equiv H_{q}^{\ln_{q}}=\frac{1}{q-1}(1-\sum_{i\in \chi }p_{i}^{q}).$$

It was introduced to model systems where standard Boltzmann–Gibbs or Shannon approaches might fail, particularly those with strong correlations, long-range interactions or fractal-like phase-space structures (e.g. turbulence and astrophysical systems). Unlike Shannon entropy—additive for independent subsystems—Tsallis entropy typically exhibits non-extensive or ‘q-additive’ behaviour. When two systems are combined, their total entropy follows $T_{q}(X,Y)=T_{q}(X)+T_{q}(Y)+(1-q)T_{q}(X)T_{q}(Y)$. Also, while Tsallis entropy converges to Shannon entropy ($\lim_{q\to 1}T_{q}(X)=H(X)$), when evaluated in the uniform distribution U, it does not retrieve Boltzmann entropy unless q=1. Instead, in general, $T_{q}(U)=(\Omega^{1-q}-1)/(1-q)$.

## 4. Case studies

A full examination of the axioms’ validity lies beyond the scope of this contribution. Instead, we present two case studies—in economics and ecology—that illustrate why these axioms matter in practice. We focus on axiom 4, in both its strong and weak forms (§2), as this distinction has the greatest practical impact.

### 4.1. Economics

For this example, we follow the formalism outlined in Caticha & Golan [21]. Consider the economies of two countries A and B that are separated by some trade barrier. Country A (respectively, B) has agents labelled $a=1,\ldots ,N_{A}$ (respectively, $b=1,\ldots ,N_{B}$), and the goods are labelled g=1,…,G. Let $f_{ag}$ denote the amount of good g produced by agent a, $\chi_{agg^{\prime }}$ denote the amount of good g used as an input for the production of good $g^{\prime }$ and $y_{ag}$ denote the amount used in general household consumption. The microstate of an economy is then a particular configuration of all inputs and consumed goods:

(4.1) $$\{\chi_{agg^{\prime }}\}\equiv x^{\left(A\right)},\;\;\{y_{ag}\}\equiv y^{\left(A\right)}\;\text{and}\;\{\chi_{bgg^{\prime }}\}\equiv x^{\left(B\right)},\;\;\{y_{bg}\}\equiv y^{\left(B\right)}.$$

Following Caticha & Golan [21], the probability distribution over the microstates of country A (and similarly of B) is obtained by maximizing the Shannon entropy subject to the following constraints on production and consumption:

— $\langle \sum_{a,g^{\prime }}\chi_{agg^{\prime }}\rangle =X_{g}^{\left(A\right)}$   (average usage of good g in production),
— $\langle \sum_{a}y_{ag}\rangle =Y_{g}^{\left(A\right)}$   (average consumption of good g), and
— $\langle \sum_{a}f_{ag}\rangle =F_{g}^{\left(A\right)}$   (average production of good g).

Because we assume a trade barrier, all consumed goods must have been produced in the focal country, so an additional constraint reads

(4.2) $$F_{g}^{\left(A\right)}=X_{g}^{\left(A\right)}+Y_{g}^{\left(A\right)},$$

reflecting that a country must ‘use up’ the goods it produces, either in further production or in consumption; an analogous relation holds for B.^1^

Writing $x_{ag}\equiv \sum_{g^{\prime }}\chi_{agg^{\prime }}$ for the total input usage and collecting the constrained quantities of each economy into the single linear combination

(4.3) $$u^{\left(A\right)}\equiv \sum_{a,g}(\alpha_{g}\;x_{ag}-\beta_{g}\;f_{ag}+\gamma_{g}\;y_{ag}),$$

with Lagrange multipliers $\alpha_{g},\beta_{g},\gamma_{g}$ (and $u^{\left(B\right)}$ defined analogously over the agents of B), the maximum-entropy solution for a single economy is the Gibbs distribution $P(x^{\left(A\right)},y^{\left(A\right)})\propto e^{-u^{\left(A\right)}}$, in which the endogenous prices appear as the combinations $\pi_{g}=\frac{1}{2}(\alpha_{g}+\gamma_{g})-\beta_{g}$. Now, if we treat the two countries as separate systems and impose only independent constraints for each, this solution factorizes over agents [21], and the joint distribution of the two economies is simply the product

(4.4) $$P(x^{\left(A\right)},y^{\left(A\right)},x^{\left(B\right)},y^{\left(B\right)})=P(x^{\left(A\right)},y^{\left(A\right)})\;P(x^{\left(B\right)},y^{\left(B\right)})\;\propto \;e^{-u^{\left(A\right)}-u^{\left(B\right)}}.$$

This factorization is a direct consequence of the SSI axiom in Shannon’s framework (§2).

However, real-world economies frequently display correlations that do not vanish merely because trade barriers or other constraints appear to isolate them. For example, both countries still share the same planet’s finite resources.^2^ A disruption in one region’s resource extraction may ripple into other regions. Geopolitical events (wars, pandemics or financial market crashes) can simultaneously affect production and consumption in each country, creating correlations even when there is no active trade. Finally, indirect spillover effects (through innovation, global supply chains and environmental impacts) can correlate the two economies, even if those interactions are not captured by explicit constraints.

In other words, the absence of explicit ‘cross-country’ constraints in the optimization does not necessarily mean the two subsystems are truly independent; it simply means one has not explicitly modelled their interdependence. By using Shannon entropy subject only to separate constraints, one unavoidably loses any hidden correlation—Shannon’s SSI enforces factorization.

On the other hand, generalized entropies (e.g. Rényi, Tsallis or other non-Shannon forms) relax the assumption of SSI and thus allow for the possibility that correlations might exist or emerge naturally, even if the constraints do not explicitly couple the countries.

Hence, if one has a strong reason to suspect subtle interdependencies—such as resource limits, global shocks or other correlated phenomena—but can only enforce ‘independent’ constraints in one’s data-fitting procedure, a generalized maximum-entropy framework might be more faithful to the underlying reality. It can accommodate correlations that remain hidden under a purely Shannon-based treatment.

There are two ways to accommodate correlations that one suspects but cannot, or does not wish to, write down as an explicit coupling. It is instructive to contrast them on the joint distribution (4.4).

*Route 1: a generalized entropy, with the same constraints.* Generalized entropies—Rényi, Tsallis or other non-Shannon forms (§3)—relax SSI and thereby allow correlations to be present even when the constraints do not explicitly couple the countries. Concretely, maximizing such an entropy under the identical constraints replaces the exponential in equation (4.4) by the q-exponential, $\exp_{q}(z)\equiv [1+(1-q)z{]}^{1/(1-q)}$, giving

(4.5) $$P(x^{\left(A\right)},y^{\left(A\right)},x^{\left(B\right)},y^{\left(B\right)})\;\propto \;\exp_{q}\;(-u^{\left(A\right)}-u^{\left(B\right)}).$$

Unlike the ordinary exponential, the q-exponential is not multiplicative; exactly,

(4.6) $$\exp_{q}(u)\;\exp_{q}(v)\;=\;\exp_{q}\;(u+v+(1-q)\;uv)\;\neq \;\exp_{q}(u+v)\;(q\neq 1).$$

Consequently, equation (4.5) does not factorize into a function of A’s microstate times a function of B’s: the two economies are statistically coupled even though no cross-country constraint was imposed. Comparing equation (4.5) to the product of the two separate solutions makes the induced dependence explicit,

(4.7) $$\exp_{q}\;(-u^{\left(A\right)})\;\exp_{q}\;(-u^{\left(B\right)})\;=\;\exp_{q}\;(-u^{\left(A\right)}-u^{\left(B\right)}+(1-q)\;u^{\left(A\right)}u^{\left(B\right)}),$$

so the coupling is carried by the term $(1-q)\;u^{\left(A\right)}u^{\left(B\right)}$ and vanishes precisely when q=1, recovering the Shannon factorization. In economic terms, the multipliers still price the individual quantities, but their contributions to −log⁡P no longer add: the shadow value of a bundle acquires a non-extensive^3^ correction of order 1−q.

*Route 2: the Shannon entropy, with an added coupling constraint.* Alternatively, one may keep the Shannon entropy but introduce a constraint that explicitly couples the two economies—for instance a constraint on the cross-country covariance $\langle A^{\left(A\right)}A^{\left(B\right)}\rangle =c$ of two aggregate outcomes. The solution then acquires an explicit interaction term, $P\propto e^{-u^{\left(A\right)}-u^{\left(B\right)}-\nu \;A^{\left(A\right)}A^{\left(B\right)}}$, and is non-factorized by construction, with the coupling strength ν a freely tunable multiplier. This is exactly the kind of ‘additional constraint’ that Caticha & Golan [21] note is required to introduce such interactions into their framework in the first place.

The two routes correspond to genuinely different modelling situations. Route 2 requires one to know which interaction to model and to be able to express it as a constraint; it then returns that interaction, quantified by ν. Route 1 requires no such knowledge: it leaves the constraints separate and lets a single parameter q absorb the departure from independence. In practical economic modelling, one often lacks complete or precise information on all the ways two countries might influence one another, one may have no direct data on shared resource limits, spillover effects or global shocks. When one nonetheless has good reason to believe that SSI is an unrealistic assumption, route 1 is the more careful approach: even when only separate constraints can be reliably enforced, a generalized maximum-entropy framework retains enough flexibility to capture correlations that Shannon’s factorization would discard, and is in this sense more faithful to the underlying reality. This is precisely the broader point made by Jizba & Korbel [11]: lacking explicit information about interactions does not imply their absence.

*Relation to quantal response statistical equilibrium (QRSE).* Route 2 isolates the mechanism behind the QRSE models of Scharfenaker & Foley [23] and their applications to firm-size [24,25] and labour-market [26] distributions, in which dependence is introduced through a covariance constraint within the Shannon programme rather than by changing the entropy. QRSE supplies the behavioural microfoundation that route 2 leaves abstract: agents make discrete choices (enter or exit, expand or contract) according to a logit quantal response derived from a satisficing constraint, and the model is closed by a feedback constraint that fixes the covariance between an agent’s *action and the aggregate outcome* [26], an intra-system coupling, in contrast to the cross-system coupling of route 2. Our framework and QRSE are thus two *orthogonal* enrichments of the same Shannon baseline: QRSE holds q=1 and adds constraints, shaping the body of the distribution and setting the rate of its exponential tails, whereas relaxing SSI keeps the constraints minimal and moves q away from unity, changing the tail *class* from exponential to power law. Neither family contains the other, and they coincide only where a change of variable matches the two tail classes. The practical implication is a division of labour: where a behavioural coupling can be named and a response variable identified, the Shannon-plus-covariance route is preferable and returns interpretable behavioural parameters; where the coupling is unknown, or the correct variable differs between the body and the tail of the distribution, relaxing SSI substitutes a single learnable parameter q for a mechanistic specification one may not possess. We develop this comparison in detail in appendix B.

### 4.2. Ecology

Consider a forest where two separate inventories, A and B, have been carried out. Location A (respectively B) has species labelled $s_{a}=1,\ldots ,S_{A}$ (respectively $s_{b}=1,\ldots ,S_{B}$). The relative abundance of species i in plot A is $p_{A}(i)=n_{A,i}/N_{A}$, where $n_{A,i}$ is the number of individuals of species i recorded in A and $N_{A}=\sum_{i}n_{A,i}$ the total number of individuals there; $p_{B}(i)$ is defined analogously for B. There is some overlap in the species found in either location but the abundances vary and each site also has unique species.

If we assume that the two plots are truly independent owing to spatial separation, following the SSI axiom 4b, the total entropy of the forest based on these samples would simply be the joint entropy given by the sum of their individual entropies (H(A,B)=H(A)+H(B)). SI fails, however, if the species abundance distributions (SADs) in location A and B are not independent, for example, by shared environmental characteristics or historical reasons of which we might not even be aware. The independence fails because knowing something about location A would already give information on what to expect for location B. In this case, we should account for what is termed the shared information between A and B as they are now no longer simply additive, defined as: H(A,B)=H(A)+H(B)−I(A;B). The last term in this equation is termed mutual information, representing the degree of dependence between the two systems. The mutual information between two systems can be stated as the marginal entropy of A minus the conditional entropy of A given what we know about B:

(4.8) $$\begin{array}{rl} I(A;B) & =H(A)-H(A|B)\\ & =H(B)-H(B|A), \end{array}$$

given by $\sum_{i,j}p_{AB}(i,j)\log\frac{p_{AB}(i,j)}{p_{A}(i)\;p_{B}(j)}$, H(A) and H(B), respectively, for the separate marginal entropies of plot A and B, H(A|B) or H(B|A) for the conditional entropy of either A or B taking into account B or A and finally H(A,B) for the joint entropy of the two plots taken together.

From this set of equations, it is clear that if there is no shared information (i.e. I(A;B)=0, so that knowing one plot does not reduce the uncertainty about the other and H(A|B)=H(A)) the entropy of the forest as a whole is indeed the two separate entropies combined. If there is, however, shared information, then I(A;B)>0, and entropy is no longer strictly additive as specified in the Shannon functional form. The total entropy of the system should then be corrected for the mutual information.

A simple example to illustrate the consequence of violating the SSI axiom is when we try to estimate the diversity of the forest by calculating the so-called *effective* number of species. Introduced by MacArthur [27] in 1965, for Shannon entropy measures of diversity, this is given by $S_{\text{eff}}=e^{H}$. If we assume hypothetically that for location A, $H(A)=-\sum_{i}p_{A}(i)\log p_{A}(i)=1.8$ and for location B, $H(B)=-\sum_{i}p_{B}(i)\log p_{B}(i)=1.4$, two scenarios are possible: either there is no mutual information, I(A;B)=0 or that I(A;B)>0. In the former case, the effective number of species is then given by $S_{\text{eff}}=e^{H(A)+H(B)}$, while in the latter $S_{\text{eff}}=e^{H(A)+H(B)-I(A;B)}$. If locations A and B are not independent, the mutual information could be positive, e.g. I(A;B) could be 0.34. If we then take these numbers, it is clear that we would overestimate the number of effective species if we did not take into account these dependencies:

(4.9) $$\begin{array}{r} \text{uncorrected:}\;S_{\text{eff}}=e^{1.4+1.8}\approx 25\\ \text{and corrected:}\;S_{\text{eff}}=e^{(1.4+1.8)-.34}\approx 18. \end{array}$$

Thus, if the overlap is ignored and SI is assumed, the combined entropy (and therefore diversity) of the forest will be overestimated as one does not account for the shared information between the two systems. Often we lack information about possible (historical) interactions between the two plots. In that case, applying the maximum-entropy procedure in the *Shannon* functional form under independent constraints forces the joint probability to factorize, because SSI is built into the Shannon form. That factorization sets I(A;B)=0 by construction, and when the two plots are in fact correlated it leads to an overestimation of the effective number of species.

A generalized entropy approach however can handle violations of SSI by using other functional forms of the entropy maximization, like the Rényi or Tsallis entropy which offer a more flexible framework to account for potential interdependencies, e.g. less or more emphasis on dominant species for examples that are shared between the two plots.

The same functional—Shannon entropy, say—can play two distinct roles, and the axiomatic concerns of this paper attach to only one of them. As an *inference functional*, used within the MEP to construct probability distributions, the choice of entropy is a modelling assumption: employing Shannon entropy implicitly enforces SSI, and it is in this role that an inappropriate choice may introduce systematic bias if that assumption is violated. In such cases, generalized entropy functionals provide a principled way to relax these assumptions during inference. As a *descriptive statistic*, by contrast, a summary computed from a distribution one already has, such as Shannon diversity used to quantify evenness or richness within a given sample, no axiom is at stake. In both cases (Shannon or generalized entropies), the MEP is a tool to construct probability distributions that are maximally non-committal with respect to missing information. Once a joint distribution has been inferred, standard information-theoretic quantities, including Shannon entropy or mutual information, may then be used as descriptive measures of diversity or dependence, without inconsistency. In this latter case, entropies become a tool to scout different properties of the system, such as Shannon diversity or Rényi diversity (which assigns different weights to the tail of the distribution).

## 5. Simple model

This section introduces a minimal model that violates SSI. A signature of this violation is the sub-exponential scaling of the phase space (number of feasible configurations) as a function of the number of degrees of freedom. Intuitively, this indicates the presence of strong correlations among the constituents of the system that make the SSI axiom non-applicable (§6).

Consider a one-dimensional chain consisting of N binary correlated random variables (bits), taking values $s_{i}\in \{0,1\}$. Although abstract, binary random variables are a general description that can capture many applications. For example, the ‘chain’ can be considered as a list of species found in a sample where the presence or absence of a species (i.e. 1 or 0) is or is not dependent on the presence or absence of the other species (i.e. the other variables in the chain), conditional on whether we are dealing with a system satisfying SSI or not. Hereafter, we refer to the chain of binary random variables as a general form.

Taking inspiration from Ruseckas [28], we consider that bits next to each other have almost always the same value, but there are d cases (‘flips’) where the next bit has a different value. For example, if N=10 and d=2, the sequence ‘0011111100’ is allowed, with flips in positions 2 and 8. This rule effectively decreases the number of allowed configurations; in particular, while an unconstrained chain of bits’ phase space scales exponentially with respect to N, in this case, it scales as a power of N, precisely as $\Omega_{d}(N)∼(2/d!)N^{d}$.

By calling $M=\sum_{i}^{N}s_{i}$ the sum of the chain (i.e. the total number of species corresponding to the value $s_{i}=1$) from [28], we also know the number of configurations leading to a given sum M, for N>>d:

(5.1) $$\Omega_{d}(N,M)=\frac{N^{d-1}}{\kappa (d)}{\left(1-4\frac{(M-N/2{)}^{2}}{N^{2}}\right)}^{\left\lfloor \frac{d-1}{2}\right\rfloor },$$

where $\kappa (d)=d^{d-2}\lfloor \frac{d}{2}\rfloor !\lfloor \frac{d-1}{2}\rfloor !$ and ⌊⌋ is the floor function.

Our objective is to infer the least-biased distribution over configurations $c=\{s_{1},\ldots ,s_{N}\}$. We therefore apply the generalized maximum-entropy approach. We maximize the Rényi entropy,

(5.2) $$R_{q}[P]=\frac{1}{1-q}\;\ln\;\sum_{c}p(c{)}^{q},$$

which §3 identifies as the member of the family that keeps maximum entropy consistent with maximum likelihood, and which tends to Shannon entropy as q→1. The maximization is carried out under normalization and one further constraint, which fixes the q-mean of M,

(5.3) $$\langle M\rangle_{q}\equiv \frac{\sum_{c}p(c{)}^{q}\;M(c)}{\sum_{c}p(c{)}^{q}}=M^{*},$$

the mean of M under the escort distribution $p(c{)}^{q}/\sum_{c^{\prime }}p(c^{\prime }{)}^{q}$, which equals the ordinary mean at q=1 [18, eqn (31)]. Constraining the q-mean, a nonlinear functional of p, rather than an ordinary mean is what allows the inferred description to factorize at a coarse (ensemble) scale while retaining dependence at finer scales; in Shore–Johnson terms this corresponds to relaxing subset independence, as expected for nonlinear constraints [18].

Introducing multipliers α and θ for the two constraints, the stationary points of the Lagrangian

(5.4) $$L_{q}[P]=R_{q}[P]-\alpha (\sum_{c}p(c)-1)-\theta (\langle M\rangle_{q}-M^{*})$$

(their eqn (32)) satisfy $\partial L_{q}/\partial p(c)=0$, which gives a q-exponential (their eqns (33)–(39)):

(5.5) $$p_{q}(c,\theta )\;\propto \;[\;1-(1-q)\;\theta \;(M(c)-\langle M\rangle_{q})\;{]}_{+}^{1/(1-q)},\;(x{)}_{+}^{a}\equiv \left\{ \begin{array}{clclc} & x^{a}, & \; & x>0, & \\ & 0, & \; & x\leq 0. & \end{array}\right.$$

Equation (5.5) is not yet explicit, because the q-mean $\langle M\rangle_{q}$ that the constraint fixes also appears on its right-hand side. Following Somazzi and Garlaschelli, we absorb this dependence into the multiplier by setting

(5.6) $$\psi (\theta )\equiv \frac{\theta }{1+(1-q)\;\theta \;\langle M\rangle_{q}}$$

(their eqn (41)), which brings the distribution to the closed form used throughout:

(5.7)p(c)=1Zq(ψ)[1−(1−q)ψM(c)]+1/(1−q)(5.8)andZq(ψ)=∑c[1−(1−q)ψM(c)]+1/(1−q)=∑M=0NΩd(N,M)[1−(1−q)ψM]+1/(1−q).

The reparametrized multiplier ψ is not an independent quantity: it carries the same information as θ and reduces to it as q→1, the limit in which p(c) becomes the Boltzmann–Gibbs distribution $e^{-\theta M(c)}/Z_{1}(\theta )$.

For illustration, figure 1 shows the bare q-exponential $p_{q}(x)\propto [1-(1-q)\psi x{]}_{+}^{1/(1-q)}$ for a single constraint C(x)=x at different values of q and a common scale ψ in linear axes and log–log axes. The family splits into three regimes: for q<1, the density has finite support, vanishing beyond $x_{\max}=1/[(1-q)\psi ]$; for q=1, it is the Shannon exponential; for q>1, it develops a power-law tail of exponent −1/(q−1), heavier as q grows. Intuitively, the presence of strong correlations increases the probability of high values of M.

This power-law tail also fixes the choice of constraint. For q>1, the q-exponential decays as a power law, so its ordinary mean need not exist: ⟨M⟩ is finite only for q<3/2 and diverges for 3/2≤q<2, even though the distribution is normalizable throughout q<2. The q-mean $\langle M\rangle_{q}$, by contrast, stays finite across the whole range (appendix C). Since q is not known before the fit, we cannot assume the ordinary mean exists, which is why the constraint in equation (5.3) is placed on the q-mean rather than the ordinary mean; as the inference below shows, this is also the choice that keeps the maximum-likelihood estimate of q consistent with the entropy.

**Figure 1.**
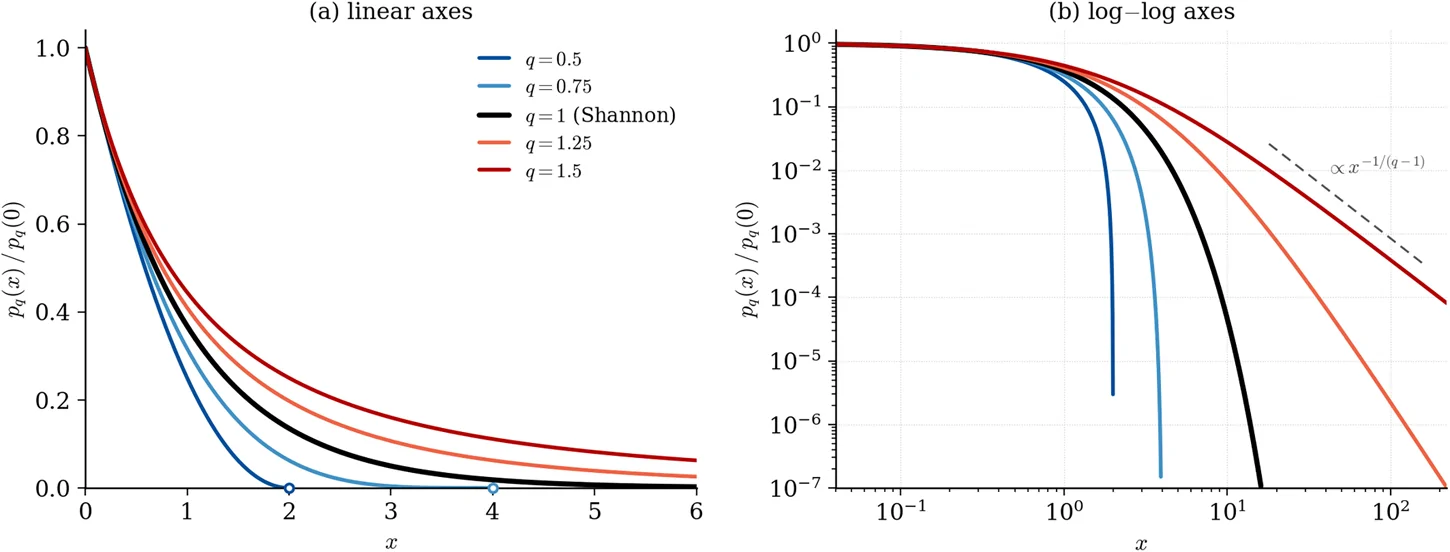
The q-exponential $p_{q}(x)\propto [1-(1-q)\psi x{]}_{+}^{1/(1-q)}$, shown normalized to its peak so that all curves meet at x=0 (C(x)=x, ψ=1). Colour runs from blue (q<1) through black (the Shannon case q=1) to red (q>1). (a) Linear axes: the curves coincide near the origin—the body is shared to first order—and separate only in the tail; for q<1, the support is finite, the density vanishing at $x_{\max}=1/[(1-q)\psi ]$ (open circles), while for q>1, it decays more slowly than the exponential. (b) Log–log axes: for q>1, the tail is a power law of slope −1/(q−1) (dashed guide), heavier as q grows; for q=1, it is exponential; for q<1, it is cut off at $x_{\max}$. The entropic index interpolates from compactly supported (q<1) through exponential (q=1) to heavy-tailed (q>1), acting only on the tail.

Let us now focus on the estimation of parameters from observations,^4^ as detailed in [18] (see box 3 for a quick recap and appendix C for a more complete derivation). Given a series of K independent observations^5^ $\vec{M^{*}}=\{M_{1}^{*},\ldots ,M_{K}^{*}\}$, we would like to maximize the log-likelihood of our data with respect to the parameters q and ψ:

(5.9) $$\nabla_{\left(q^{*},\psi^{*}\right)}\log\prod_{k=1}^{K}p(c_{M_{k}^{*}}|q,\psi )=\vec{0}.$$

The resulting $\left(q^{*},\psi^{*}\right)$ is consistent and asymptotically efficient, though, like maximum-likelihood estimators in general, biased at finite sample size. We run a simple numerical experiment, considering a chain of length N=100 and flips d=4. We sampled ${\vec{M}}^{*}=\{M_{1}^{*},\ldots ,M_{1000}^{*}\}$ values to learn our parameters, according to a power-law distribution. The resulting values are $\left(q^{*},\psi^{*})\approx (1.821,1.337\right)$, meaning that our inference procedure suggests a different entropy and thus a non-exponential distribution (q≠1). Figure 2 a shows the histogram of the observed ${\vec{M}}^{*}$ and the overlapped inferred distribution $p(M|q^{*},\psi^{*})$. Figure 2b shows a contour plot of the log-likelihood surface, expressed as the difference $\ell_{\text{max}}-\ell (q,\psi )$ between the maximum value ($\ell_{\text{max}}=\ell (q^{*},\psi^{*})$) and the value at each (q,ψ). This re-centring sets the optimum at zero and highlights how quickly the likelihood declines away from the maximum, a standard approach that also connects directly to likelihood-ratio confidence regions.

**Figure 2.**
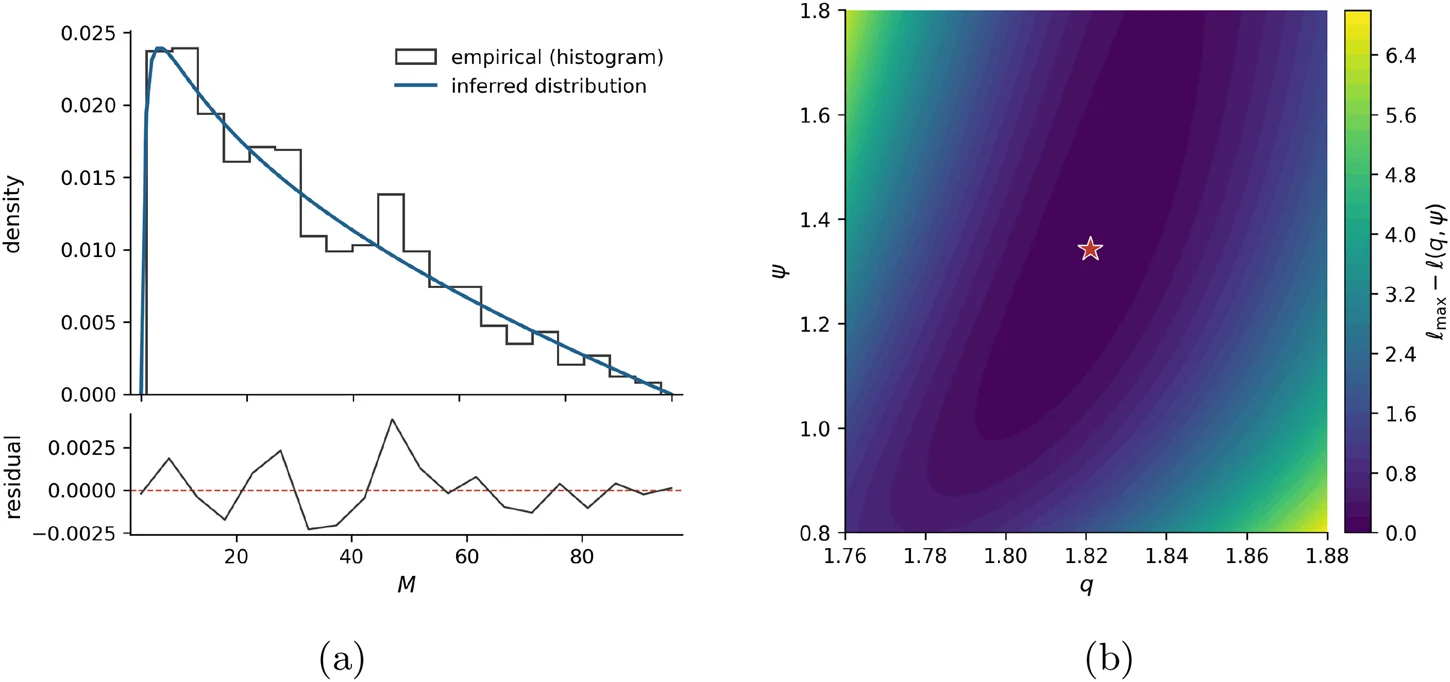
(a) Histogram of observed ${\vec{M}}^{*}$, together with inferred probability distribution $p(M|q^{*},\psi^{*})$. (b) Contour plot of the log-likelihood surface, expressed as the difference $\ell_{\text{max}}-\ell (q,\psi )$ between the maximum value (at the maximum likelihood estimator $\left(q^{*},\psi^{*}\right)$) and the value at each (q,ψ).

**Box 3. Learning the entropic parameter from data**

**Problem.** Let the system take microstates x∈Ω, each carrying an observable (or vector of observables) C(x); in the chain model of this section, $C(x)=M=\sum_{i}s_{i}$. Given K i.i.d. realizations $D=\{x^{\left(k\right)}{\}}_{k=1}^{K}$ with values $C^{\left(k\right)}:=C(x^{\left(k\right)})$, determine both the *least-biased* distribution over the microstates and the value of the entropic index q that the data support.

**Solution.** Maximizing the Rényi entropy (5.2) under normalization and the q-mean constraint (5.3) gives the q-exponential ( [18, eqns (42) and (43)], and our appendix C)

$$p_{q}(x,\psi )=\frac{[1-(1-q)\;\psi \;\cdot \;C(x){]}_{+}^{1/(1-q)}}{Z_{q}(\psi )},$$

with $Z_{q}(\psi )$ the normalization and ψ the reparametrized multiplier (5.6). It remains to fit $\left(q^{*},\psi^{*}\right)$ by maximum likelihood.

**Step 1: optimize ψ for fixed q.** For each trial, q maximizes

$${\bar{\ell }}_{q}(\psi )=\frac{1}{K}\sum_{k=1}^{K}\;\ln p_{q}(x^{\left(k\right)},\psi )$$

to obtain $\psi_{q}^{*}$. Stationarity gives the q-mean matching rule

$$\sum_{x\in \Omega }\;C(x)\;p_{q}^{\;q}(x,\psi_{q}^{*})=\frac{1}{K}\sum_{k=1}^{K}C^{\left(k\right)}\;p_{q}^{\;q-1}\;(x^{\left(k\right)},\psi_{q}^{*}).$$

**Step 2: select q.** With ψ fixed by step 1 at each q, the log-likelihood becomes a function of q alone; its maximizer is the estimate,

$$q^{*}=\arg\max_{q}\;{\bar{\ell }}_{q}(\psi_{q}^{*}),\;\psi^{*}=\psi_{q^{*}}^{*}.$$

A coarse grid in q with a one-dimensional refinement suffices in practice. Because Rényi is the entropy that reconciles maximum entropy with maximum likelihood (§3), this $q^{*}$ is fixed by the data, not chosen by hand.

**Practical suggestions.**

— Newton or limited-memory BFGS for $\psi_{q}^{*}$ (one solve per q); Brent or a log-parabola search over q.
—
$q^{*}=1$ recovers the classical recipe of box 1; $q^{*}\neq 1$ signals a violation of SSI.
— Heavy-tailed samples need larger K before $q^{*}$ stabilizes.

The example above shows that by using a generalized maximum-entropy approach, together with a maximum-likelihood estimation procedure [18], the ‘correct’ entropy emerges from the data. In this case, data indicated that SSI is violated and Shannon entropy would be inapplicable. If it were not the case, the maximum-likelihood procedure—provided sufficient amount of data—would retrieve the Shannon case ($q^{*}=1$). The same run lets us compare against Shannon quantitatively. Fixing q=1 and optimizing over ψ alone gives ψ(q=1)≈0.0432 and a maximized log-likelihood ℓ(1,ψ(1))≈−14159, against $\ell (q^{*},\psi^{*})\approx -14\;071$ for the free-q fit. The two models are nested and differ by a single parameter, so the gap of roughly 88 in log-likelihood translates into ΔAIC≈174 and ΔBIC≈169 in favour of q≠1, with a likelihood-ratio statistic 2Δℓ≈176 on one degree of freedom ($p\approx 10^{-40}$). Had the phase space grown exponentially instead, the same fit would have returned $q^{*}=1$ given enough data.

Finally, figure 3 shows how the inferred parameters depend on the chain length N, the number of flips d and the number of realizations n. While the example discussed above used N=100, d=4 and n=1000, here we repeat the inference over a range of values of these parameters, performing R=300 independent repetitions for each parameter combination. When one parameter is varied, the other two are held fixed at the reference values of that example (for instance, when varying d we set n=1000 and N=100). The top row shows the inferred q and the bottom row the inferred ψ, while each column corresponds to a different varied parameter (n, N and d). Each grey dot is a single inferred value, the shaded blue band spans the interquartile range (25th–75th percentile), and the blue markers indicate the median. In the bottom row, the red percentages give the fraction of estimated ψ that exceed the (arbitrarily chosen) threshold ψ=3 and therefore lie outside the plotted range.

**Figure 3.**
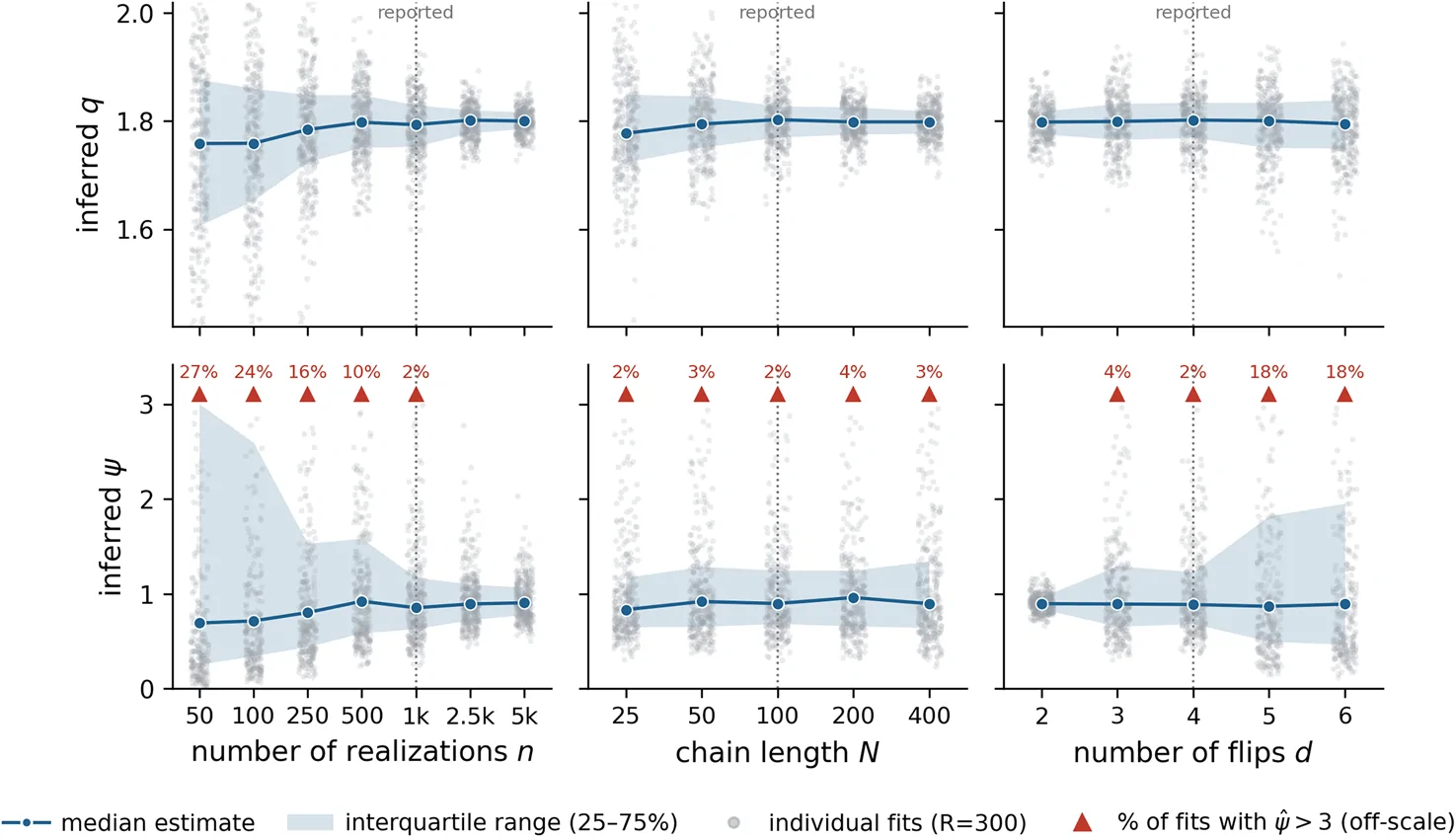
Sensitivity of the inferred parameters to the chain length N, the number of flips d and the number of realizations n. Estimates are obtained from R=300 independent synthetic samples fitted with the same procedure as in the main text. In each column, one parameter is varied while the other two are fixed at the reference configuration N=100, d=4 and n=1000 (dotted vertical line). Top row: inferred q; bottom row: inferred ψ. Grey dots are individual estimates, the blue band is the interquartile range (25th–75th percentile) and the blue markers denote the median. In the bottom row, the red percentages report the fraction of $\hat{\psi }$ that exceed the arbitrary threshold ψ=3 and thus fall outside the displayed range. The precision of both estimates improves markedly with n, is largely insensitive to N, and degrades as d increases.

As expected, the spread of the estimates shrinks as the number of realizations increases. The chain length, by contrast, has only a weak influence on the inference, because the latter relies on the ‘magnetization’ M, an aggregate quantity. A larger number of flips, however, increases the variance of the estimates: as d grows, the average size of an island of aligned spins decreases, which narrows the range of accessible magnetizations—in the limit d=N−1, the magnetization is pinned to its central value M=N/2 (zero net magnetization). This progressive loss of variability in M makes the parameter estimates noisier. To complete the picture, we verify that the procedure recovers the generating distribution itself. We draw synthetic samples (n=1000, R=200 repetitions) from the model at a range of known entropic indices $q_{\mathrm{true}}$, holding the scale fixed at $\psi_{\mathrm{true}}=0.05$ so that the distribution stays well spread across the range, and re-estimate $\left(q^{*},\psi^{*}\right)$ from each sample. Figure 4 shows that the inferred $q^{*}$ tracks $q_{\mathrm{true}}$ across all three regimes, compactly supported (q<1), exponential (q=1) and heavy-tailed (q>1). In particular, when the data are generated at $q_{\mathrm{true}}=1$, the estimator returns $q^{*}\approx 1$, recovering the Shannon case rather than spuriously preferring q≠1. A mild downward bias and a widening spread appear for $q_{\mathrm{true}}≳1.6$, where the tail is the heaviest and a fixed sample carries less information about q, the finite-sample bias expected of maximum-likelihood estimators. The scale $\psi^{*}$ is likewise recovered around its true value. The inferred parameters thus reflect the sampling distribution as intended: $q^{*}$ identifies the tail class of the data-generating process rather than being an artefact of the fit.

**Figure 4.**
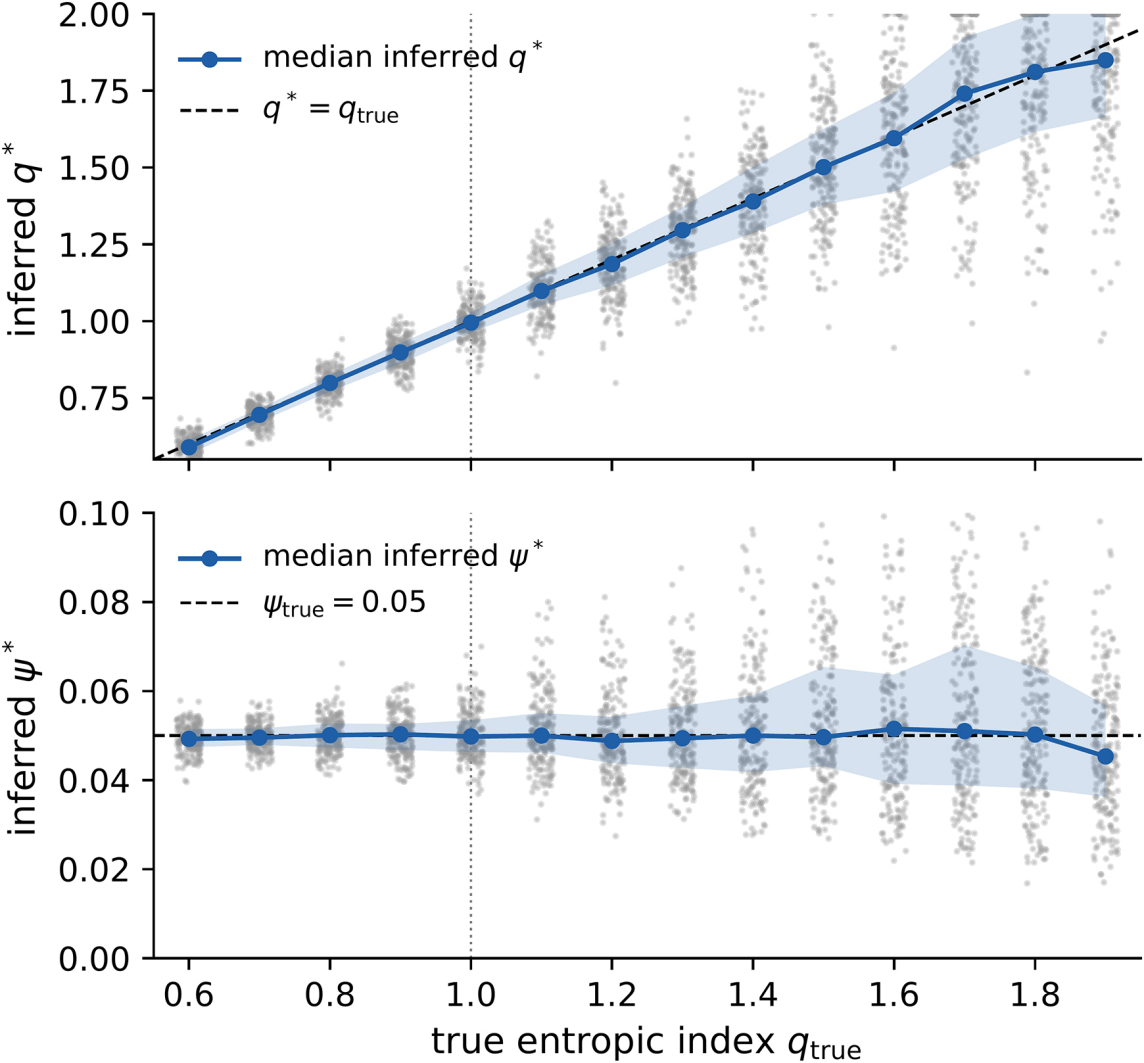
Parameter recovery across generating distributions. Synthetic samples (N=100, d=4 and n=1000) are drawn from the model at known $\left(q_{\mathrm{true}},\psi_{\mathrm{true}}=0.05\right)$ and re-fitted; each point aggregates R=200 independent repetitions. Top: inferred $q^{*}$ versus $q_{\mathrm{true}}$; the dashed line is exact recovery ($q^{*}=q_{\mathrm{true}}$) and the dotted vertical line marks the Shannon case $q_{\mathrm{true}}=1$. Bottom: inferred $\psi^{*}$; the dashed line is $\psi_{\mathrm{true}}$. Grey dots are individual fits, blue markers are the median and the shaded band is the interquartile range. The estimator recovers $q_{\mathrm{true}}$ across the compact, exponential and heavy-tailed regimes, returning $q^{*}\approx 1$ for Shannon-distributed data; the mild bias and growing spread for $q_{\mathrm{true}}≳1.6$ reflect the finite-sample bias of maximum likelihood in the heavy-tailed regime.

## 6. Discussion and future outlook

The MEP procedure using Shannon entropy has found countless applications in a diverse array of disciplines. Simultaneously, there has been a considerable debate on the applicability of Shannon entropy given the underlying assumptions or axioms associated with it. Given the importance of these axioms for inferential purposes, there exists a body of research that explores the use of more generalized entropy functions—such as Rényi or Tsallis entropy—as flexible alternatives for situations that violate some of the foundational assumptions of classical entropy. Much of it is, however, primarily of a theoretical nature and a more intuitive explanation was missing. Here, we have discussed these axioms, their implications and the challenges they bring, with a specific emphasis on the axiom of SI to illustrate some of the consequences and bring the concept of generalized entropy to a wider audience.

The major challenge we consider is that the classic Shore–Johnson axiomatic derivation of the MEP assumes implicitly SSI axiom: whenever two subsystems are disjoint, one may impose constraints on each separately or on the joint system interchangeably. Imposing SSI forces the entropic functional to factorize additively and thereby uniquely singles out Shannon entropy as the only viable inference measure. In such case, a bias arises when SSI is violated, owing to the fact that Shannon forces factorization and discards correlations; when SSI holds, Shannon is correct. There are, however, many alternatives to the functional form of the entropy maximization and when we take a more general look we see that Shannon entropy is simply part of a larger (one-parameter) family of entropies. In fact, if SSI is not imposed, the Shore–Johnson consistency still holds for the broader Uffink–Jizba–Korbel (UJK) class of functionals for the maximization of entropy given in its general form by

$$U_{q}(P)\;\propto \;(\sum_{i}p_{i}^{q}{)}^{1/(1-q)},$$

modulo any monotonic transformation. In other words, relaxing SSI yields a range of one-parameter entropies, with Shannon as the q→1 limit. The question that arises is then for any practical applications: when do we expect the SSI criterion to fail?

*Phase-space growth of a complex system*. A concrete criterion for the validity of SSI is the scaling of the phase-space volume W(N) with system size N. The phase-space volume of a system is defined here as the number of admissible microstates, i.e. all possible configurations of the constituent elements consistent with the imposed macroscopic constraints. As the size of the system increases, the number of possible configurations grows; what matters, however, is the manner in which this growth scales with N.

For systems composed of effectively independent subsystems, the phase space grows exponentially with system size (box 2), so that log⁡W(N) is extensive. A canonical example is a system of N independent binary elements (or, equivalently, an unbiased random walk with two choices at each step): each added element doubles the number of accessible configurations, yielding $W(N)=2^{N}$. In this case, the assumptions underlying SSI apply, and Shannon entropy is appropriate.

When SSI fails, structural constraints alter the scaling of the admissible configuration space, rendering log⁡W(N) non-extensive. A simple illustration is provided by a correlated (or ‘ageing’) random walk [29], in which successive choices constrain increasingly large blocks of steps to proceed in the same direction. Although each elementary choice remains binary, correlations restrict the set of admissible paths, and the number of configurations grows sub-exponentially rather than as $2^{N}$. In this case, large classes of configurations are forbidden by construction, and additivity in logarithmic space no longer holds.^6^ In mathematical terms, if

$$W(N)∼e^{c_{0}N},$$

then subsystems remain effectively independent and Shannon entropy (q=1) applies. Whenever the scaling of W(N) deviates from this exponential form, whether through sub-exponential restriction or super-exponential proliferation of admissible configurations, SSI fails, and one should instead employ a generalized entropy with q≠1. The scaling of W(N) with system size thus provides a principled diagnostic for the validity of SSI.

The phase-space criterion also bears on a natural concern: when a generalized entropy produces a non-factorizing joint distribution under independent constraints, does this reflect genuine structure in the system, or merely an artefact of the entropy one has chosen? A maximum-entropy distribution does not by itself identify the entropy that generated it—any monotonically increasing function of an entropy is maximized by the same distribution [18], so fitting a maximum-entropy form to data and reading off q is not, on its own, evidence that q≠1. We therefore do not treat a departure from Shannon as a default; it must be supported by evidence external to the choice of functional, and two independent routes are available. The first is structural: the scaling of W(N) is a property of the system’s admissible configuration space, and sub- or super-exponential growth is precisely the regime in which SSI is unjustified [11]. The second is empirical: once the entropy form is fixed—the requirement that the entropy of the uniform distribution not depend on q selects Rényi within the UJK family and places all its members on a common scale [18]—the value of q is estimated by maximum likelihood, as in §5. That estimate is identifiable only from informative, non-uniform data (a uniform sample carries no information about q), and it is singled out by a self-consistency condition: at the maximum-likelihood optimum the Shannon entropy of the fitted distribution equals minus its log-likelihood (appendix C and equation (C 14)), an identity that a value of q chosen by hand would not satisfy.

Both routes return q=1, and hence factorization, whenever the structure and the data are compatible with independence, and depart from it only on positive evidence. Genuine structure is therefore indicated when q≠1 is fixed by non-exponential phase-space scaling or by informative-data maximum likelihood with this self-consistency holding, while model-dependent non-factorization is what results when q is imposed by hand. Two caveats delimit the interpretation. The entropy so identified is an information-theoretic inference functional and need not coincide with the system’s physical entropy [18]; what the procedure guarantees is that the inferred *distribution* is the least-biased description consistent with the data. The resulting non-factorization is not the assertion of a particular interaction, but a refusal to *assume* independence where the structure or the data do not warrant it. In other words, a dependence that is absent across independent replicas at the ensemble scale yet may be present within a system at finer scales, with q the single parameter that carries it.

The diagnostic should not be overstated into a selection rule. The scaling of W(N) indicates *whether* SSI holds, and hence whether a generalized entropy is needed, but it does not by itself fix *which* functional, or which value of q, to use in an inference. In the sample-space scaling classification of Korbel *et al.* [29], the scaling exponents of the configuration space do determine the entropy, but only up to a class of asymptotically equivalent functionals, and only by treating the entropic parameter as structural knowledge of the system, supplied separately from the empirical data [18]. In a maximum-entropy *inference* setting, this sits uneasily with the requirement that the inference remains maximally noncommittal, which asks that q, like the other parameters, be determined by the data rather than fixed by external assumption. In our framework, the functional and the value of q are therefore fixed by inference rather than by the scaling (a consistency requirement sets the form, and q is estimated by maximum likelihood (§5)). Phase-space scaling and the inferred q thus play distinct but mutually consistent roles: the scaling diagnoses that SSI fails, the data quantify by how much and their agreement is a check rather than one determining the other.

*Interpreting the entropic index.* Because q is the single parameter that distinguishes a generalized entropy from the Shannon case, it is natural to ask what it means physically. There is no universal answer: across systems, q admits several compatible readings, all measuring the departure from the independent, extensive baseline at q=1. It quantifies non-extensivity, or equivalently how the phase-space volume W(N) departs from exponential growth [20,29]; it sets the relative weight of rare versus common states, and hence the tail class of the distribution, which in ecology is the order of a Hill-number diversity profile [16,17]; in superstatistics, it measures the fluctuations of an intensive parameter (such as a fluctuating inverse temperature) across sub-ensembles, with q−1 equal to the relative variance of that parameter and q=1 in its absence [30]; in particular systems, it is fixed by measurable observables, as when Jizba & Korbel [11] relate it to the heat capacity of a hadronic heat bath. In the inference setting used here, q is first an information-theoretic index determined by the data; a physical interpretation requires an additional, system-specific mapping. Accordingly, q is best reported as an inferred quantity and interpreted through the mechanism of the system at hand, as in our chain model, where q reflects the sub-exponential phase space induced by the flip constraint, or in the ecological application discussed below [31], where the inferred q tracks soil-chemistry gradients.

*A scientist’s guide to entropies*. Given these considerations, we provide a step-by-step procedure that we believe should ensure proper use of the MEP:

1. Explore the dimensionality of the system under study, i.e. what constitutes the potential phase space of the system, and what are the underlying structures of the probability distributions over which maximization of entropy will be applied? In this step, it is important to get a more conceptual perspective of the potential caveats.
2. Analytically diagnose the scaling of W(N) (e.g. via sample-space enumeration, fitting to various functional forms of scaling or empirical partition-function fits).
  — If $W(N)\propto e^{O(N)}$, stick with Shannon entropy.
  — Else, adopt the generalized entropy $U_{q}(P)$ and, rather than fixing q beforehand, treat it as a free parameter estimated jointly with the constraint multipliers by maximum likelihood (box 3 and [18]). This procedure is self-correcting: the fitted q returns to 1, recovering Shannon entropy, precisely when W(N) grows exponentially, and departs from 1 only when it does not.
3. Visualize different outcomes of using various forms of the entropy families by adjusting the q-parameter and general entropy profiles.
4. Decide on entropy measure and outline considerations in methodology for reproducibility and accountability.

*When phase space is inaccessible*. In some applications, however, the diagnostic in step (2) cannot be carried out directly, because the underlying microstate space is either unknown or deliberately coarse-grained. Two practical routes then remain. When the state space can still be sampled or partially enumerated, the growth of W(N) need not be obtained in closed form: one can fit candidate functional forms to the accessible portion of the state space and map the resulting scaling onto the corresponding universality class [29], so that the step (2) diagnostic is carried out approximately rather than exactly. When even this is out of reach, one can dispense with W(N) altogether. Because the scaling only diagnoses whether SSI holds and does not itself select the entropy, and because the entropic index is ultimately fixed by the data, one may instead posit the distributional form directly, typically from a small set of macroscopic state variables, and estimate q from the observations by maximum likelihood, exactly as in §5. Full knowledge of the microstate space is thus not a prerequisite for the inference; what is required is only enough structure to write down the likelihood of the observed data.

The inaccessibility of the phase space arises, for example, in macroecology, the study of large-scale patterns in the distribution of abundances and body sizes of organisms across species and spatial scales. A prominent framework in this field is the maximum entropy theory of ecology (METE) [32], which derives community-level patterns, such as the SAD and species–area relationship, from a small set of state variables (e.g. total abundance and total metabolic energy), without specifying an explicit individual-level microstate space. In its standard formulation, METE adopts Shannon entropy, although Harte himself explicitly noted the possibility that more general entropy functionals may be appropriate for ecological systems with internal elements exhibiting long-range interactions, memory effects or far-from-equilibrium dynamics [33].

A recent extension of METE operationalizes this idea by replacing the Shannon functional with Rényi entropy and treating the entropic parameter q as an unknown quantity to be inferred from data [31]. Starting from a Rényi–MaxEnt derivation of the SAD, the authors estimate q by maximum likelihood, fitting the predicted abundance distribution to observed species abundances within individual forest plots subject to the usual METE state-variable constraints. In this likelihood-based procedure, the set of observed species abundances within a given plot is treated as the collection of repeated observations: each species’ abundance constitutes one data point drawn from the predicted Rényi–METE SAD. This likelihood-based identification of the entropic parameter is formally identical to the procedure described in §5, where generalized MaxEnt models are embedded within a standard maximum-likelihood framework. Applied to 49 tropical forest plots in Panama, the inferred values of q vary substantially across sites (ranging approx. from 1/2 to 1) and exhibit statistically significant associations with soil-chemistry variables, most notably phosphorus (negative association with q) and aluminium and ammonium (positive associations). The authors interpret these patterns in terms of how soil-chemistry structures species responses: phosphorus is argued to have heterogeneous, species-specific effects that amplify dominance and reduce evenness, whereas aluminium and ammonium act as more uniformly suppressive or homogenizing stressors across taxa.

## 7. Conclusion

Although our analyses of (complex) systems can be greatly aided by tools from information theory, it also brings with it the requirement of more abstract notions of how we think of these systems. In essence, it requires a more probabilistic point of view and with it the associated rules of engagement related to calculations of probabilities. From an applied standpoint, nothing fundamentally changes about the choice of constraints when moving from Shannon to a generalized entropy: one still conditions on expectation values, solves via Lagrange multipliers, and only afterwards determines those multipliers (and q itself) by fitting to observed averages. There is, however, a deeper consideration necessary that we have emphasized here that we believe is critical if we are to derive meaningful conclusions. We hope this conceptual overview provides some of the insights necessary for practitioners to consider the limitations of these methods but also their power to analyse real-world systems.

## Data Availability

The code used for this work is available at: http://github.com/Peppe95/Generalized_Maximum_entropy_when_why and has been archived within the Zenodo repository [34].

## Footnotes

^1^
Caticha & Golan [21] additionally impose constraints on agents’ expected utilities and budgets; we omit these here, as they play no role in the factorization argument that follows.

^2^
The link between entropy and the finiteness of an economy’s resource base has a long lineage. Georgescu-Roegen [22] argued that the economic process is ultimately constrained by the (thermodynamic) entropy law, so that low-entropy matter and energy, not produced goods as such, constitute the ultimate scarce resource. That argument is thermodynamic rather than information-theoretic, and is thus complementary to the inferential use of entropy we adopt here.

^3^
The exact form of this correction depends on whether the constraints are imposed as ordinary or as escort averages, but the non-factorization for q≠1 does not, since it follows solely from equation (4.6).

^4^
The power-law scaling of $\Omega_{d}(N,M)$ allows for non-trivial distributions, and for the analytical solution of $Z_{q}(\psi )$ (and therefore of other quantities such as averages like ⟨M⟩) for some parameters’ values.

^5^
We imagine that such observations come from controlled repeated experiments, meaning that we know they are independent of each other, and thus their joint probability factorizes into the product of the marginals.

^6^
Strong correlations can also produce the opposite effect, leading to super-exponential growth of the phase space. An example is given by systems of interacting (‘magnetic’) binary elements, where interactions between constituents generate new composite states in addition to the original ones. In such cases, the number of accessible configurations grows faster than exponentially with N.

## Appendix A. Formal statement of system independence

We give here the formal counterpart of §2.4, in the maximum-entropy operator formalism used by Uffink [13], Jizba & Korbel [11] and Somazzi & Garlaschelli [18]. Let ∘I denote the distribution that maximizes the chosen entropy S subject to a constraint set I (with uniform prior). For two systems A,B with state spaces $\Omega_{A},\Omega_{B}$, let $I_{A},I_{B}$ be constraints involving only A and only B (no coupling term) and $I_{A}\wedge I_{B}$ their conjunction on $\Omega_{A}\times \Omega_{B}$; write $p_{A}=\circ I_{A}$, $p_{B}=\circ I_{B}$ and $p_{AB}=\circ (I_{A}\wedge I_{B})$.

*SSI* (axiom 4b) requires the joint inference to factorize, for every separable pair $I_{A},I_{B}$:

(A 1) $$p_{AB}(x,y)=p_{A}(x)\;p_{B}(y).$$

*SI* (axiom 4a) requires only *consistency*: inferring A and B separately or jointly must never conflict. Uffink [13] proved that this requirement (with axioms 1–3 and 5) holds if and only if the entropy belongs to the family $S=f(U_{q})$, $U_{q}(P)=(\sum_{i}p_{i}^{\;q}{)}^{1/(1-q)}$ of equation (3.1). For that family, Jizba & Korbel [11] show that the joint and separate solutions obey

(A 2) $$[\;p_{AB}\;U_{q}(p_{AB})\;{]}^{q-1}-1\;=\;([\;p_{A}\;U_{q}(p_{A})\;{]}^{q-1}-1)\;+\;([\;p_{B}\;U_{q}(p_{B})\;{]}^{q-1}-1)$$

(their eqn (8)), a q-deformed composition that is the concrete content of the consistency SI demands.

Equation (A 2) collapses to the product (A 1) if and only if q=1—equivalently, if and only if the entropy is strictly additive, S[AB]=S[A]+S[B]—that is, only for Shannon entropy. SSI is therefore strictly stronger than SI: a product is one particular consistent composition, so equation (A 1) implies equation (A 2), and the two coincide precisely at q=1. For every other member of the family (q≠1), the maximum-entropy joint is not a product, and A and B stay correlated even though $I_{A}$ and $I_{B}$ do not couple them. This is the precise sense in which independent constraints are not the same as independent systems, and it is why relaxing 4b to 4a preserves Shore–Johnson consistency for the whole family while admitting entropies beyond Shannon.

## Appendix B. The generalized-entropy framework and quantal response statistical equilibrium compared

The QRSE models of economics [23–26] and the framework of this paper are both constrained maximum-entropy models fitted to the same data. They differ in two respects: the entropy functional and the constraints imposed. This appendix makes the comparison precise for a one-dimensional outcome variable x, and identifies similarities and differences. Throughout, we write $\exp_{q}(z)=[1+(1-q)z{]}^{1/(1-q)}$ for the q-exponential and $\sigma (z)=(1+e^{-z}{)}^{-1}$ for the logistic function.

### B.1. The quantal response statistical equilibrium approach

A QRSE model describes an outcome variable x∈R, such as a profit rate, a firm’s log size and a wage, that emerges from the individual *quantal actions* a of many interacting agents. In the binary case, the action is a choice between two alternatives, a∈{enter,exit} (or expand/contract, accept/reject). The model is the joint distribution f(a,x), whose two conditional distributions encode a theory of the interaction [23]: f(a∣x), the probability of an action given the outcome (the behavioural response of agents to x), and f(x∣a), the effect of the actions on the outcome (competition). The response f(a∣x) is taken to be the logit σ((x−μ)/T), which is itself the maximum-entropy mixed strategy subject to a minimum-expected-payoff, or *satisficing*, constraint; here, T is a behavioural scale (a ‘temperature’) and μ a threshold. The interaction is closed by a *feedback* constraint linking actions back to the outcome, which is equivalent to fixing the covariance between the action and the outcome [26]. Maximizing the Shannon entropy of f(a,x) subject to normalization, a constraint on the mean outcome (with multiplier γ) and the feedback constraint $\int \tanh(\frac{x-\mu }{2T})\;x\;f(x)\;\text{d}x\leq \delta$ (with multiplier β) yields the marginal

(B 1) $$\begin{array}{rl} f_{Q}(x) & \;\propto \;e^{H_{T,\mu }(x)}\;\exp\;[-\gamma x-\beta \;\tanh(\frac{x-\mu }{2T})\;x]\\ \text{and}\;H_{T,\mu }(x) & =-\;\;\sum_{a}f(a|x)\log f(a|x), \end{array}$$

where $H_{T,\mu }(x)$ is the binary entropy of the logit response. The multipliers γ and β are internal to the QRSE program and are unrelated to the economics multipliers of §4.1. The resulting shape is a sharply peaked, exponentially tailed distribution: the one-sided model gives a tent-shaped (Laplace- or Subbotin-like) profit-rate or firm-size distribution, and the two-sided labour-market model can in addition be skewed or platykurtic. Its tails are exponential in x, with right rate γ+β and left rate β−γ: as x→±∞, $H_{T,\mu }(x)\to 0$ and $\tanh(\frac{x-\mu }{2T})\to \pm 1$, so $-\log f_{Q}(x)\to (\gamma +\beta )x$ on the right and (γ−β)x on the left; a normalizable two-sided density requires β>|γ|, with γ setting the skew.

### B.2. Similarities and difference between the approaches

In contrast to QRSE, the generalized-entropy approach introduces no agents, no actions and no joint distribution f(a,x). It models the distribution of the outcome directly—or, more precisely, the distribution over the system’s microstates, of which the outcome is a function—by maximizing a generalized entropy of that distribution subject to a single constraint on an observable C(x). Where QRSE builds the outcome distribution up from a behavioural model of individual actions, the generalized approach specifies it directly, through a choice of entropy and one constraint. The constraint is placed on the escort (q-)mean: the escort (or ‘zooming’) distribution is $\tilde{p}(x)\propto p(x{)}^{q}$, and one fixes $\langle C\rangle_{q}=\int C(x)\;p(x{)}^{q}\;\text{d}x\;/\;\int p(x{)}^{q}\;\text{d}x$. Taking the natural unbounded, asymptotically linear observable C(x)=x on x≥0, the counterpart of the QRSE mean constraint, the maximum-entropy density is the q-exponential, which for 1<q<2 normalizes to

(B 2) $$\begin{array}{rl} p_{q}(x) & =\psi (2-q)\;[1+(q-1)\psi x{]}^{-1/(q-1)}\\ \text{and}\;{\overset{―}{P}}_{q}(x) & \equiv \int_{x}^{\infty }\;p_{q}(x^{\prime })\;\text{d}x^{\prime }=[1+(q-1)\psi x{]}^{-\rho },\;\rho =\frac{2-q}{q-1}, \end{array}$$

where ψ>0 is the multiplier of the constraint and ${\overset{―}{P}}_{q}$ is the survival function (complementary cumulative distribution) with a power-law tail of index ρ.

Over a fixed outcome variable, then, the QRSE marginal is exponentially tailed while the q-exponential is power-law tailed, and the two parametric families coincide only in their common exponential limit (β,T→0 for QRSE and q→1 for the q-exponential). However, note that the q-exponential is *itself* a Shannon maximum-entropy density, but for a nonlinear feature rather than for x. Writing equation (B 2) as $p_{q}\propto e^{-\lambda \varphi_{q}(x)}$ with λ=1/(q−1) and

(B 3) $$\varphi_{q}(x)=\log\;[1+(q-1)\psi x],$$

one recovers equation (B 2) exactly, and expanding the logarithm shows that $\varphi_{q}(x)\approx (q-1)\psi x$ for small x and $\varphi_{q}(x)\approx \log x$ for large x, with the change-over at $x^{⋆}=1/[(q-1)\psi ]$. A single q-exponential is therefore the Shannon maximum-entropy density that imposes a mean constraint on the body of the distribution and a logarithmic (geometric-mean) constraint on its tail. Equivalently, at the level of the modelled variable, applying QRSE to log⁡X instead of X turns its exponential tail into a power-law tail in X. Both approaches can thus produce power-law tails.

What separates them lies in what must be supplied and what is inferred. QRSE encodes the dependence between actions and outcomes in an explicit constraint of a specific functional form—the feedback covariance $\left\langle \tanh(\frac{x-\mu }{2T})\;x\right\rangle$, motivated by a behavioural mechanism—and then estimates the behavioural parameters (T,μ,γ,β). The generalized program supplies no such mechanism: it imposes a single generic constraint (the q-mean of C) and absorbs the departure from an extensive, independent description into the entropic parameter q, which is itself estimated from the data. This is precisely the situation Somazzi & Garlaschelli formalize [18], in which the observations come from replicas of a system that are independent at the coarse (ensemble) scale but may carry correlations at finer scales, a hierarchical, scale-dependent dependence that q captures with a single parameter rather than with a specified coupling.

This difference in what each program constrains—QRSE the ordinary mean (and the feedback covariance), and the generalized program the escort q-mean—carries a further, concrete consequence, in the very existence of those quantities. If the tail in the modelled variable is heavier than ρ≤1—that is, $q\geq \frac{3}{2}$ in equation (B 2)—the ordinary mean $\int x\;p_{q}(x)\;\text{d}x$ diverges, so the quantity QRSE constrains is not estimable and the law of large numbers it relies on fails. The escort mean, by contrast, is $\left\langle x\rangle_{q}=1/[\psi (2-q)\right]$, finite for every q∈(1,2): the generalized constraint remains well defined precisely where the QRSE constraint breaks down. However, as reported earlier, a monotonic transformation changes the tail class, i.e. the logarithm of a Pareto variable is exponentially tailed with a finite mean, which is again why size studies model log-size [25]. In other words, what the generalized program offers is the ability to adapt the tail class without having first identified the correct linearizing transformation.

### B.3. A worked illustration: the firm-size distribution

Suppose one observes a cross-section of a non-negative size variable X with a heavy right tail—firm sales, say [24,25]—and wishes to infer its equilibrium distribution. The two programs handle the same sample $\left\{x_{i}\right\}$ differently.

Following QRSE, one commits to a state variable and a behavioural mechanism. Taking the state to be Y=log⁡X, as in [25], and imposing the constraints of §B.1 gives a marginal that is exponentially tailed in Y at rate γ+β (equation (B 1)). Transforming back to $X=e^{Y}$, the induced law is a *double-Pareto* distribution—a power law on both sides, with right tail index $\rho_{Q}=\gamma +\beta$ and an interior mode when the left rate exceeds unity.

Following the generalized program, one models the raw variable X directly with C=x, obtaining the monotonic q-exponential (B 2) with a single power-law tail of index $\rho_{G}=(2-q)/(q-1)$.

The two inferred laws share the same right-tail index if and only if

(B 4) $$\gamma +\beta =\frac{2-q}{q-1},$$

but they agree only there. Their bodies are diagnosably different: the QRSE law, back-transformed, is double-Pareto, with a power-law approach to the origin and a possible interior mode, whereas the q-exponential is monotonic, with finite density at x=0 and a single tail. A practitioner therefore discriminates between the two by the body and the small-x behaviour of the data, not by the tail.

In practice, the choice between the two depends on whether one seeks a behavioural account of the data. If the object of interest is a mechanism, a behavioural temperature, a feedback strength, or the implied action probabilities, then QRSE is the appropriate model, and it is well suited to data with a sharp interior peak or a skewed or flat-topped body on a centred variable, since its covariance constraint (and, in the two-sided case, a second threshold) generates exactly these shapes, which a one-parameter q-exponential cannot. If instead one seeks only a faithful description of a heavy-tailed, unimodal quantity, a size or wealth distribution with a rounded body and a power-law tail, the generalized program is the more economical choice: it recovers the tail class and the body-to-tail change-over from the data, without requiring the correct variable to have been identified in advance, and it remains applicable even when the raw-variable mean diverges. When the correct response variable is already known, QRSE fitted in that variable yields both the tail and the mechanism, and the generalized entropy adds nothing. Note that, because the two families are non-nested, they should be compared not by a likelihood-ratio test but by a non-nested test, such as Vuong’s, or by an information criterion that penalizes the difference in the number of parameters.

## Appendix C. Derivation of the generalized maximum-entropy inference

The distribution and the estimation procedure used in §5 and box 3 are derived here, so that the argument is self-contained. We follow Somazzi & Garlaschelli [18], recast in the notation of the present paper. Throughout, x∈Ω labels a microstate carrying an observable C(x) (for the chain of §5, $C(x)=M=\sum_{i}s_{i}$), $p_{x}$ is the probability of x, and all sums run over Ω.

### C.1. The generalized maximum-entropy distribution

We maximize the Rényi entropy

(C 1) $$R_{q}[P]=\frac{1}{1-q}\ln\sum_{x}p_{x}^{\;q}$$

subject to normalization $\sum_{x}p_{x}=1$ and to a constraint on the q-mean,

(C 2) $$\langle C\rangle_{q}\equiv \frac{\sum_{x}p_{x}^{\;q}\;C(x)}{\sum_{x}p_{x}^{\;q}}=C^{*}.$$

With Lagrange multipliers α (normalization) and θ (constraint), we extremize

(C 3) $$L_{q}[P]=R_{q}[P]-\alpha (\sum_{x}p_{x}-1)-\theta (\langle C\rangle_{q}-C^{*}).$$

The partial derivatives of the two functionals are

(C 4) $$\frac{\partial R_{q}}{\partial p_{x}}=\frac{q}{1-q}\;\frac{p_{x}^{\;q-1}}{\sum_{x^{\prime }}p_{x^{\prime }}^{\;q}}\;\text{and}\;\frac{\partial \langle C\rangle_{q}}{\partial p_{x}}=q\;\frac{p_{x}^{\;q-1}(C(x)-\langle C\rangle_{q})}{\sum_{x^{\prime }}p_{x^{\prime }}^{\;q}},$$

so the stationarity condition is

(C 5) $$\frac{\partial L_{q}}{\partial p_{x}}=\frac{q}{1-q}\;\frac{p_{x}^{\;q-1}}{\sum_{x^{\prime }}p_{x^{\prime }}^{\;q}}-\alpha -\theta \;q\;\frac{p_{x}^{\;q-1}(C(x)-\langle C\rangle_{q})}{\sum_{x^{\prime }}p_{x^{\prime }}^{\;q}}=0.$$

Multiplying equation (C 5) by $p_{x}$ and summing over x, the last term vanishes, because it is proportional to $\langle C\rangle_{q}-\langle C\rangle_{q}=0$, and normalization gives α=q/(1−q). Substituting α back and solving equation (C 5) for $p_{x}$ yields the q-exponential

(C 6) $$p_{x}=\frac{[\;1-(1-q)\;\theta \;(C(x)-\langle C\rangle_{q})\;{]}_{+}^{1/(1-q)}}{W_{q}(\theta )}\;\text{and}\;W_{q}(\theta )=(\sum_{x^{\prime }}p_{x^{\prime }}^{\;q}{)}^{1/(1-q)},$$

with $[y{]}_{+}^{a}=y^{a}$ for y>0 and 0 otherwise. Equation (C 6) is implicit: the constrained quantity $\langle C\rangle_{q}$ appears on its right-hand side. Introducing the reparametrized multiplier

(C 7) $$\psi (\theta )\equiv \frac{\theta }{1+(1-q)\;\theta \;\langle C\rangle_{q}}$$

eliminates $\langle C\rangle_{q}$ and gives the explicit form used in the main text:

(C 8) $$\begin{array}{rl} p_{x} & =\frac{[\;1-(1-q)\;\psi \;C(x)\;{]}_{+}^{1/(1-q)}}{Z_{q}(\psi )}\\ \text{and}\;Z_{q}(\psi ) & =\sum_{x}[\;1-(1-q)\;\psi \;C(x)\;{]}_{+}^{1/(1-q)}. \end{array}$$

As q→1, ψ→θ, $Z_{q}\to Z_{1}$ and equation (C 8) reduces to the Boltzmann–Gibbs distribution $p_{x}=e^{-\theta C(x)}/Z_{1}(\theta )$.

*Moments and the choice of constraint.* For q>1, the density (C 8) has the power-law tail $p_{x}∼C(x{)}^{-1/(q-1)}$. Its ordinary moments therefore exist only over a limited range of q: $\langle C^{n}\rangle =\sum_{x}C(x{)}^{n}p_{x}$ converges iff 1/(q−1)>n+1, that is, iff

(C 9) $$q<\frac{n+2}{n+1}.$$

In particular, the distribution is normalizable (n=0) throughout q<2, but its ordinary mean (n=1) already diverges for q≥3/2, its variance (n=2) for q≥4/3, and so on, each successive moment requiring q closer to 1. The q-mean is instead an average against the escort weight $p_{x}^{\;q}∼C(x{)}^{-q/(q-1)}$, whose first moment $\sum_{x}C(x)\;p_{x}^{\;q}$ converges iff q/(q−1)>2, i.e. iff q<2: the q-mean is finite for *every* normalizable member of the family. Constraining the q-mean rather than the ordinary mean therefore keeps the maximum-entropy problem well posed at any q for which a distribution exists—which is what is required in §5, where q is inferred rather than fixed in advance.

### C.2. Maximum-likelihood estimation of q and ψ

Let $\{x^{\left(k\right)}{\}}_{k=1}^{K}$ be K independent observations with values $C^{\left(k\right)}=C(x^{\left(k\right)})$, and let ${\bar{\ell }}_{q}(\psi )=K^{-1}\sum_{k=1}^{K}\ln p_{x^{\left(k\right)}}$ be the average log-likelihood evaluated on equation (C 8).

*The scale ψ.* Differentiating $\ln p_{x}=(1-q{)}^{-1}\ln[1-(1-q)\psi C(x)]-\ln Z_{q}(\psi )$ and using

(C 10) $$\partial_{\psi }Z_{q}(\psi )=-\sum_{x}C(x)\;[1-(1-q)\;\psi \;C(x){]}^{q/(1-q)},$$

the condition $\partial_{\psi }{\bar{\ell }}_{q}=0$ becomes, at fixed q, the q-mean matching rule

(C 11) $$\sum_{x}C(x)\;p_{x}^{\;q}=\frac{1}{K}\sum_{k=1}^{K}C^{\left(k\right)}\;p_{x^{\left(k\right)}}^{\;q-1}.$$

The left-hand side is the model’s (unnormalized) q-mean; the right-hand side is an escort-weighted empirical average. The two coincide at the true parameters because, when the data are drawn from p, the empirical average supplies an extra factor p, turning $p^{\;q-1}$ into $p^{\;q}$. Because equation (C 11) weights the data by $p^{\;q-1}$ rather than uniformly, it stays finite whenever equation (C 8) is normalizable, even when the ordinary mean of C diverges; this is why the arithmetic average is not the correct way to combine the K observations when q is unknown.

*The index q.* Maximizing jointly over (q,ψ) adds the condition $\partial_{q}{\bar{\ell }}_{q}=0$, evaluated at the ψ of equation (C 11). The closed form (C 8) implies the relation

(C 12) $$1-(1-q)\;\psi \;C(x)=[\;p_{x}\;Z_{q}(\psi )\;{]}^{1-q},$$

and substituting equation (C 12) into the q-stationarity condition reduces it (a direct calculation, given in [18]) to

(C 13) $$\sum_{x}p_{x}\ln p_{x}=\frac{1}{K}\sum_{k=1}^{K}\ln p_{x^{\left(k\right)}},$$

that is,

(C 14) $$H[P_{q^{*}}(\psi^{*})]=-\;{\bar{\ell }}_{q^{*}}(\psi^{*}),$$

where $H[P]=-\sum_{x}p_{x}\ln p_{x}$ is the Shannon entropy. At the maximum-likelihood optimum, then, the Shannon entropy of the fitted distribution equals minus its average log-likelihood. The correspondence between entropy and likelihood that holds automatically for the exponential (q=1) is thereby recovered, at the selected $q^{*}$, for the whole q-exponential family. This is the property that singles out Rényi entropy among the family of §3, and that the estimator of box 3 exploits: maximizing the likelihood over q returns the entropy index at which inference by maximum entropy and inference by maximum likelihood coincide, with $q^{*}=1$ whenever the data are compatible with SSI.
